# Memories of Bob Sim—Genius Complementologist and Cheerful Travel Companion

**DOI:** 10.3390/v13061068

**Published:** 2021-06-04

**Authors:** Reinhard Würzner, Francesco Tedesco, Peter Garred, Tom E. Mollnes, Lennart Truedsson, Malcolm W. Turner, Yngve Sommarin, Jörgen Wieslander, Mohamed R. Daha

**Affiliations:** 1 Institute of Hygiene & Medical Microbiology, Medical University of Innsbruck, 6020 Innsbruck, Austria; 2 IRCCS, Istituto Auxologico Italiano, 20145 Milan, Italy; tedesco@units.it; 3 Laboratory of Molecular Medicine, Department of Clinical Immunology, Rigshospitalet, University of Copenhagen, 2200 Copenhagen N, Denmark; peter.garred@regionh.dk; 4 Department of Immunology, University of Oslo, 0424 Oslo, Norway; t.e.mollnes@medisin.uio.no; 5 Department of Laboratory Medicine, Section of Microbiology, Immunology and Glycobiology, Lund University, 22100 Lund, Sweden; lennart@lundabo.eu; 6 Immunobiology Unit, Institute of Child Health, University College London, London WC1N 1EH, UK; m.turner89@btinternet.com; 7 Svar Life Science, 21224 Malmö, Sweden; ysommarin@gmail.com; 8 Department of Nephrology, University of Lund, 22185 Lund, Sweden; jorgen.wieslander@telia.com; 9 University Medical Centre, 2300 RC Leiden, The Netherlands; m.r.daha@lumc.nl; 10 University Medical Centre, 9700 RB Groningen, The Netherlands

Shortly before his 70th birthday, Robert B. Sim, a genius complementologist and our dear friend and travel companion, passed away. Our memories go back for more than two decades and this obituary will only focus on our relationship with Bob during these latter years. It all started with a European Union grant involving more than a dozen European scientists and a small–medium enterprise company (Wieslab, which later merged into Eurodiagnostica and then SVAR) at the beginning of this millenium. The idea for this endeavour was to create a novel and sophisticated whole complement ELISA-based assay kit, primarily designed to screen for global complement abnormalities and deficiencies. The final result of the grant was a scientific publication [1] and a commercially available complement ELISA screening kit, WIESLAB® Complement Screen. By now, this assay has almost replaced the traditional classical and alternative complement pathway assays, CH50 and APCH50, respectively, for detection of complement deficiencies and abnormalities. A former report not only focused on the role of the mastermind and exceptional manager behind that grant, Moh Daha, but also revealed the strong support he received from Bob Sim [2].

Bob Sim was clearly not only the secretary during the grant, but the person who held all the strings together and was also an influential figure for the years after the grant, as the group decided to carry on with the collaboration with the aim to look for further applications of this assay. During the latter years, the group organized several scientific symposia throughout the world (Figure 1).

Bob was either a speaker, chairperson or discussant at these international symposia (Table 1).

The complement symposium in Wuhan in May 2019, only a couple of months before that city became infamous because of a virus everybody knows nowadays, thus became our last symposium together (Figure 2).

Apart from science, the social interactions fostered friendship and many amusing incidents happened aside from the symposia and scientific discussions. In South Africa, it was delightful to watch Bob when his wife, Edith Sim, was—almost professionally—riding an ostrich, because it was obvious that he was not sure whether she would manage; obviously, she did.

During the trip to Uruguay, the entire group was present when Bob received an honorary doctorate of the University of Montevideo, Uruguay (Figure 3).

However, not only at the day of the ceremony, but actually quite often, Bob was wearing a jacket in extremely hot weather (Figure 4), and when we would ask him “Bob, aren’t you hot?” the answer was always the same “Noooo, I’m fine!”

In conclusion, during your scientific life, you may, if you are lucky, also come across colleagues you like, others you really favour a lot, and even some you love to spend your free time with—Bob was clearly the latter; not just an intelligent, helpful and pleasant colleague, but also a cheerful travel companion and a close friend to all of us. Bob, in our hearts, you will continue to travel with us.

## Figures and Tables

**Figure 1:**
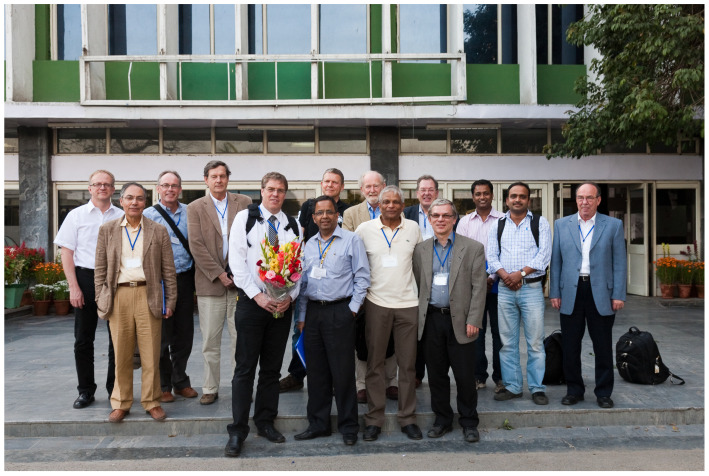
The EU complement consortium (picture taken at the teaching and research symposium in New Delhi, 27.02.10), from left to right, Yngve Sommarin (Lund), Franco Tedesco (Trieste), Jörgen Wieslander (Lund), Lennart Truedsson (Lund), Reinhard Würzner (Innsbruck), Narinda Mehra (AIIMS, New Delhi, host of symposium), Peter Garred (Copenhagen), Mac Turner (London), Moh Daha (Leiden), Bob Sim (Oxford), Tom Eirik Mollnes (Bodo), Krishana Gulla and a colleague, complementologists from Indore, and Michael Loos (Mainz), who also passed away too early. Already published in [2] with permission for re-use by Elsevier B.V., Amsterdam, The Netherlands (Ticket number 210512-012992).

**Figure 2:**
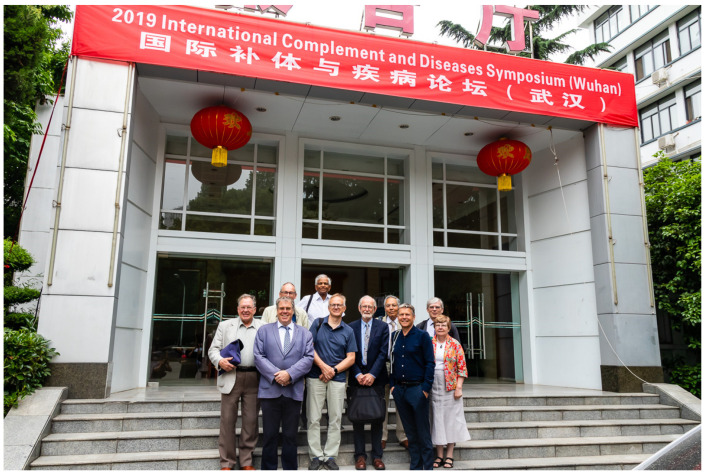
The EU complement consortium (picture taken at the International Complement and Diseases Symposium in Wuhan, 13 May 2019) with Bob (far left) making a joke so everyone was laughing, from left to right, R. Würzner, J. Wieslander, M.R. Daha (overlooking the group), Y. Sommarin, M.W. Turner, F. Tedesco, P. Garred, T.E. Mollnes and Edith Sim—not laughing; she knew the joke.

**Figure 3:**
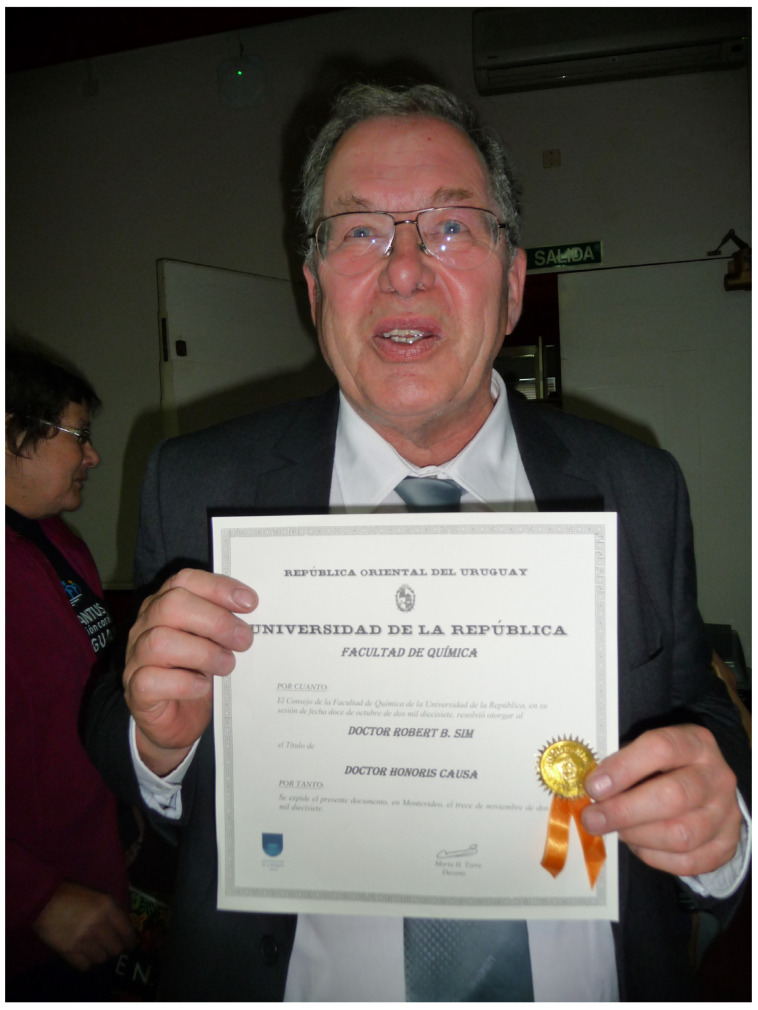
Bob Sim proudly presenting his honorary degree certificate from the Universidad de la Republica Oriental del Uruguay in Montevideo.

**Figure 4:**
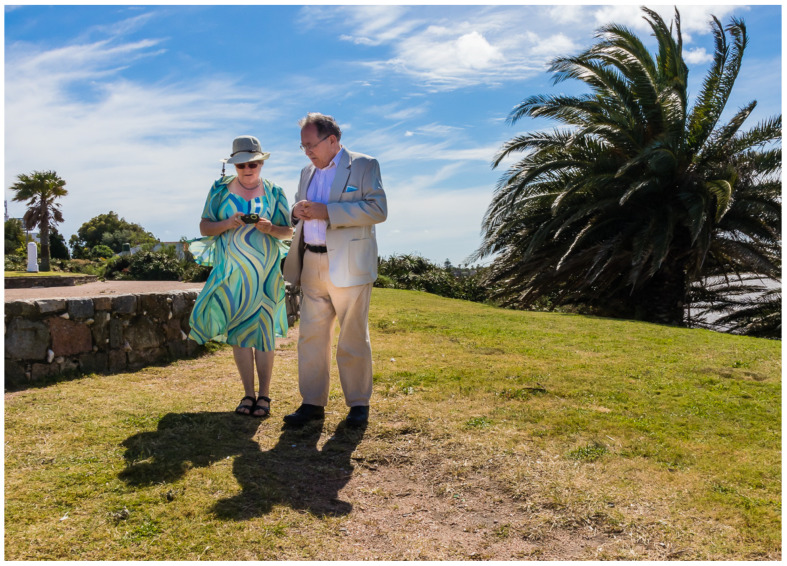
Outside Montevideo, but again properly dressed: Bob Sim with his wife Edith Sim—this photo nicely depicts the harmony between them.

**Table 1:** Scientific complement symposia and Bob Sim’s role therein.

Country	City	Date	Bob’s Role	Topic of Symposium, or Talk When Bob Was a Speaker
Surinam	Paramaribo	27.11.07	Co-Chair	Complement and Assays
India	New Delhi	27.02.10	Speaker	Quantification of Mannan Binding Lectin proteases
South Africa	Cape Town	22.11.11	Speaker	Complement and Coagulation
Sri Lanka	Colombo	26.02.14	Chair	Complement and Allergy
Vietnam	Hanoi	16.02.16	Discussant	Complement and Virus infections
Vietnam	Ho Chi Minh City	23.02.16	Discussant	Complement, HIV and Dengue
Uruguay	Montevideo	14.11.17	Honorary Speaker	Complement and Echinococcus
Argentina	Buenos Aires	21.11.17	Chair	Complement and eHUS
Hongkong	Hongkong	07.05.19	Discussant	Complement lectin pathway and Deficiencies
China	Wuhan	13.05.19	Discussant	Complement and Disease
